# Immune checkpoint inhibitor–associated myocarditis: a systematic analysis of case reports

**DOI:** 10.3389/fimmu.2023.1275254

**Published:** 2023-10-09

**Authors:** Caie Wang, Guo Zhao, Zhen Zhang, Lukui Yang, Shihao Liu, Guifang Li, Hongxia Wang, Jiaxin Huang, Shuhang Wang, Ning Li

**Affiliations:** ^1^ Department of Pharmacy, the First Affiliated Hospital of Henan University of Science and Technology, Luoyang, Henan, China; ^2^ Clinical Trials Center, National Cancer Center/National Clinical Research Center for Cancer/Cancer Hospital, Chinese Academy of Medical Sciences and Peking Union Medical College, Beijing, China

**Keywords:** immune checkpoint inhibitor, ICI-associated myocarditis, glucocorticoids, cardiovascular toxicities, case reports and series, immune-related adverse events

## Abstract

**Background:**

Immune checkpoint inhibitors (ICIs) therapy can be complicated by their potential cardiovascular toxicities, including myocarditis. Nowadays, no prospective trials have focused on ICI-associated myocarditis optimized management. Available evidence only come from case reports or series. A systematic case reports analysis was conducted to collect and evaluate emerging evidence of ICI-associated myocarditis to provide more information to clinicians.

**Methods:**

We performed a literature search for eligible case reports or series published between January 2018 and May 2023 using the PubMed database. Then, we extracted interesting information via table form. Finally, this study included 113 publications on 106 patients with ICI-associated myocarditis.

**Results:**

Myocarditis was found to be a highly life-threatening disease, with 53.8% of cases. Over half of cases were life-threatening (G4, 23.6%) or severe (G3, 35.8%) and required glucocorticoids. Higher rates of improvement were associated with the best response to ICI for complete response/partial response (72.7% vs. 53.9%), glucocorticoid administration (30% vs. 22%), and discontinuation of ICI (58.8% vs. 32.1%). Consequently, ICI-associated G3–G4 myocarditis should be treated with a combination of discontinuation of ICIs, high-dose glucocorticoids, other drugs, chemical drugs, plasma exchange, and life support. For moderate G1 or G2 cases, discontinuation of ICIs and regular-dose glucocorticoids should be considered.

**Conclusion:**

Once full recovery or improvement was achieved; glucocorticoids can be administered at low doses or stopped. Notably, re-challenge with ICIs appears feasible after resolution or meaningful improvement of myocarditis.

## Introduction

Over the past few decades, immunotherapy has revolutionized cancer treatment and has become the fourth antitumor modality after surgery, radiotherapy, and chemotherapy (1). As the frontier of cancer immunotherapy, immune checkpoint inhibitors (ICIs) have led to considerable clinical breakthroughs and extended survival rates across in a wide range of tumor types (2). ICIs are key negative regulators of antitumor immunity monoclonal antibodies, which can block immune checkpoint proteins including programmed cell death protein 1 (PD-1), programmed death ligand 1 (PD-L1), cytotoxic T-lymphocyte antigen 4 (CTLA-4), and lymphocyte activation gene 3 (LAG-3) (3). Approximately 50% of patients with cancer are eligible for ICI therapy, and a larger number of patients achieve long-term clinical responses (4). As of May 2023, 11 ICIs have been approved for marketing by the United States Food and Drug Administration (**Table 1**). The increasing number of annual clinical trials reflects the prominence of ICIs in cancer treatment (5).

**Table 1:** FDA-approved immune checkpoint inhibitors by 2023.

Types of ICIs	Drug	Approval	Sum of Indications	Indications
Anti-PD-1	Pembrolizumab	2014	18	Melanoma, Non-Small Cell Lung Cancer, Head and Neck Squamous Cell Cancer, Classical Hodgkin Lymphoma, Primary Mediastinal Large B-Cell Lymphoma, Urothelial Carcinoma, Microsatellite Instability-High or Mismatch Repair Deficient Cancer, Microsatellite Instability-High or Mismatch Repair Deficient Colorectal Cancer, Gastric Cancer, Esophageal Cancer, Cervical Cancer, Hepatocellular Carcinoma, Merkel Cell Carcinoma, Renal Cell Carcinoma, Renal Cell Carcinoma, Tumor Mutational Burden-High Cancer, Cutaneous Squamous Cell Carcinoma, Triple-Negative Breast Cancer.
	Nivolumab	2014	11	Melanoma, Non-Small Cell Lung Cancer, Malignant Pleural Mesothelioma, Renal Cell Carcinoma, Classical Hodgkin Lymphoma, Squamous Cell Carcinoma of the Head and Neck, Urothelial Carcinoma, Colorectal Cancer, Hepatocellular Carcinoma, Esophageal Cancer, Gastric Cancer, Gastroesophageal Junction Cancer and Esophageal Adenocarcinoma
	Cemiplimab	2018	3	Cutaneous Squamous Cell Carcinoma, Basal Cell Carcinom, Non-Small Cell Lung Cancer
	Dostarlimab	2021	2	Endometrial Cancer, Solid Tumors
	Retifanlimab	2023	1	Metastatic or recurrent locally advanced merkel cell carcinoma
Anti-PD-L1	Atezolizumab	2016	6	Urothelial Carcinoma, Non-Small Cell Lung Cancer, Small Cell Lung Cancer, Hepatocellular Carcinoma, Melanoma, Alveolar Soft Part Sarcoma
	Avelumab	2017	3	Merkel Cell Carcinoma, Urothelial Carcinoma, Renal Cell Carcinoma
	Durvalumab	2017	4	Non-Small Cell Lung Cancer, Small Cell Lung Cancer, Biliary Tract Cancer, Unresectable Hepatocellular Carcinoma
Anti-CTLA-4	Ipilimumab	2011	7	Melanoma, Renal Cell Carcinoma, Colorectal Cancer, Hepatocellular Carcinoma, Non-Small Cell Lung Cancer, Malignant Pleural Mesothelioma, Esophageal Cancer
	Tremelimumab	2022	2	Hepatocellular Carcinoma, Non-Small Cell Lung Cancer
Anti-LAG-3	Relatlimab	2022	1	Unresectable or Metastatic Melanoma

PD-1, programmed cell death protein 1; PD-L1, programmed cell death ligand 1; CTLA-4, cytotoxic T lymphocyte-associated antigen-4; LAG-3, lymphocyte activation gene 3.

Given that ICIs can inhibit T cells and activate immune responses, they can cause immune-related adverse events (irAEs) in any organ (6). Although any organ system can be implicated by ICI-associated irAEs, ICI-associated myocarditis has aroused as a rare and often fatal adverse event (7). Other cardiovascular toxicities include vasculitis, pericarditis, and arrhythmias (8). Timely diagnosis and proper treatment of cardiovascular irAEs, especially ICI-associated myocarditis, are clinically challenging (9). Although uncommon (<1% of patients with cancer are treated with ICIs) (10, 11), the morbidity of ICI-associated myocarditis is probably underestimated with inconsistent screening criteria and nonspecific symptoms. In clinical practice, cardiovascular irAEs may manifest occasionally; this view may attribute to the poor understanding of the disease and the failure to recognize early symptoms (12). However, ICI-associated myocarditis has a high death rate, ranging from approximately 20% to 50%, according to retrospective studies (11, 13). Inconsistent morbidity and mortality of ICI-associated myocarditis reflect an unmet clinical need; therefore, understanding the precise mechanisms of pathogenesis and having more clinical information of ICI-associated myocarditis are crucial for timely diagnosis and treatment.

Recently, some authoritative recommendations have been specifically established for the diagnosis and treatment of ICI-associated myocarditis, such as 2022 ESC Guidelines on cardio-oncology (14), Myocarditis in the Setting of Cancer Therapeutics (15). Besides, management of ICI-associated myocarditis can be found in the *Guidelines of Immune-Related Adverse Events in Patients Treated with Immune Checkpoint Inhibitor Therapy* published by the American Society of Clinical Oncology (16). However, no prospective trials have focused on ICI-associated myocarditis’ optimized management, and the available evidence was case reports or series. With the high mortality of ICI-associated myocarditis, a timely diagnosis and management is necessary to decrease the death rate and increase the application scope of ICIs in cancer patients. Therefore, we conducted a systematic analysis of case reports for the purpose of collecting and evaluating emerging evidence of ICI-associated myocarditis to provide more information to clinicians.

## Materials and methods

### Search strategy

**Figure 1:**
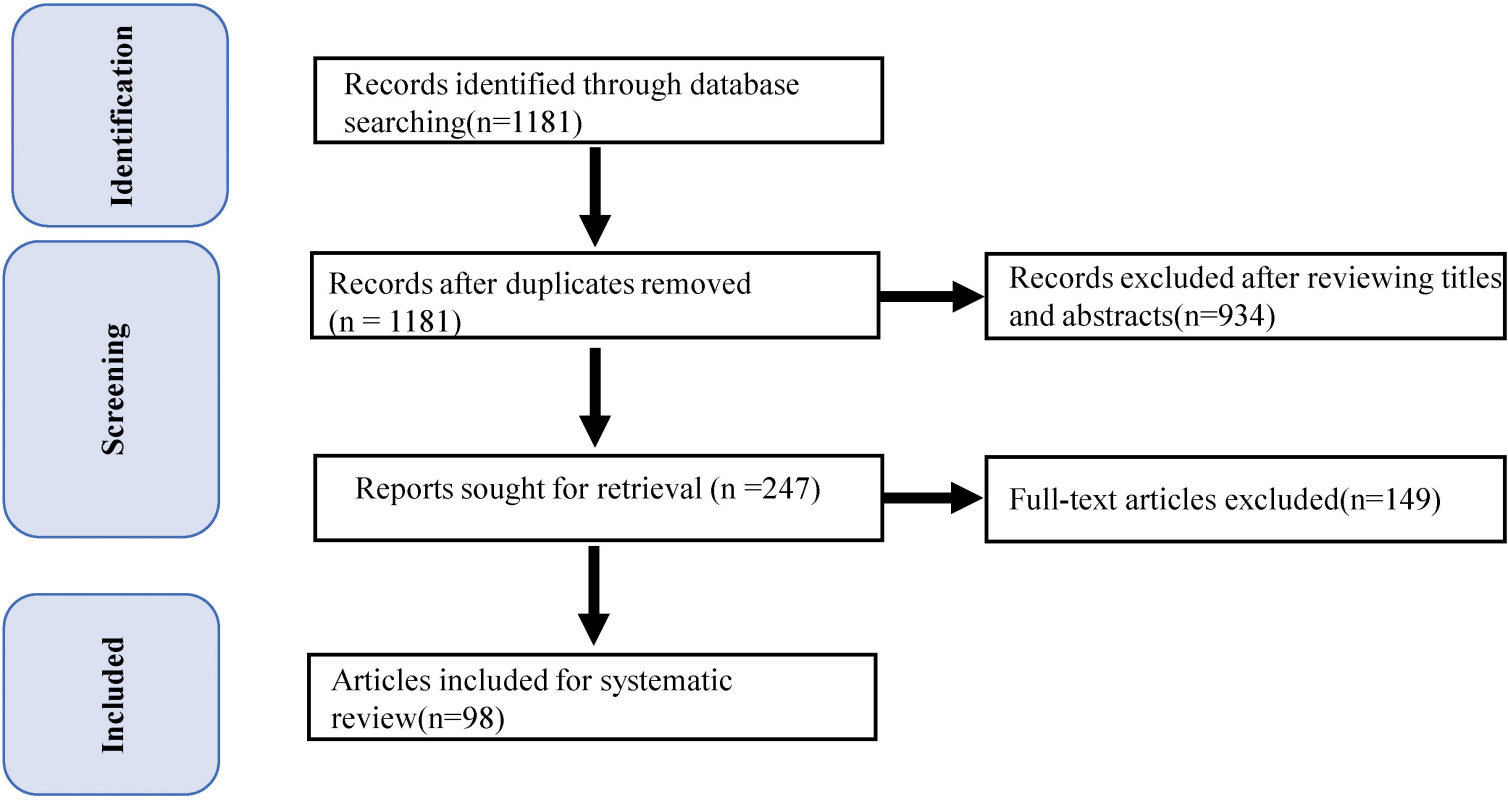
PRISMA Participant Flow Diagram. Flow diagram depicts the: 1) methods and results for our strategies; and 2) results of record screening and inclusion in the systematic review of case reports and series.

We first performed a literature search for eligible case reports or series published between January 2018 and May 2023 using the PubMed database. Then, we carried out a further search using the following combination of terms: (‘checkpoint inhibitors’ OR ‘checkpoint inhibition’ OR ‘checkpoint blockade’ OR ‘PD1’ OR ‘PDL1’ OR ‘CTLA4’ OR ‘sintilimab’ OR ‘pembrolizumab’ OR ‘camrelizumab’ OR ‘nivolumab’ OR ‘tremelimumab’ OR ‘ipilimumab’ OR ‘atezolizumab’) AND (‘carditis’ OR ‘myocarditides’ OR ‘myocarditis’ OR ‘cardiac adverse event’ OR ‘cardiac side-effect’ OR ‘cardiac toxicity’ OR ‘cardiac complication’ OR ‘cardiac irAE’ OR ‘Heart Failure’). A detailed flowchart of the study is shown in **Figure 1**.

### Eligibility criteria

Case reports were selected by preliminarily assessment of titles and abstracts. For searching additional qualifying papers, the reference lists of the included literature were curated manually. The inclusion criteria were as follows: (1) studies on ICI-associated myocarditis; (2) full-text available; (3) published papers; and (4) case reports or case series. The exclusion criteria were as follows: (1) articles, reviews, commentaries, and meta-analyses; (2) articles not written in English; (3) studies on cancer agents other than ICIs; and (4) no myocarditis studies.

### Study selection and quality assessment

All studies were independently evaluated through the Rayyan platform by screening titles and abstracts with three individuals in parallel (17). The authors assessed the studies based on the aforementioned eligibility criteria and any disagreements were resolved by a third reviewer. The quality assessment of this article of case reports was conducted by previous study. Sufficient quality was determined if five of the eight evaluation criteria were met and all authors agreed that the study could be included.

### Data extraction

For the included studies, three authors manually retrieved and extracted the related data. Details were extracted from each case report as follows: reference information (reference tittle, first author, year); basic patient information (sex, age, past medical history, cancer type, and cancer stage); ICI treatment information (ICI treatment type, therapy line, and ICI drug name); ICI-associated myocarditis information (time to onset, myocarditis diagnosis and staging, myocarditis symptoms, best response to ICI, and prognosis); and other relevant information (ICI discontinuation type, ICI-associated myocarditis treatment strategies, treatment outcome, ICI re-challenge, ICI-associated myocarditis recurrence, and associated irAEs). All data were extracted and compiled into an online Excel file with accessible permissions to all the authors.

The data extracted from each article are summarized and presented in **Supplementary Material**. The cases will be described narratively, combine and highlight the similarities between them, if possible, draw conclusions. Considering the abstractibility of this article and the small cases loads, we used descriptive statistics to exhibit the demographic and clinical characteristics of these cases. Continuous variables were reported by means, and dichotomous variables were characterized by frequencies and percentages.

## Results

The search strategy identified 1181 records, all of which were screened based on titles and abstracts. Ultimately, 98 publications were selected, including 116 cases of ICI-associated myocarditis. A descriptive summary of these 106 cases is presented in **Supplementary Material**.

**Table 2** provides a summary of the main characteristics of patients. The median time to onset of myocarditis was 22.5 days (IQR 16–52) following the initiation of ICI treatment. However, some cases of early toxicity and late toxicity are noted within the first week (18, 19) and after ≥1 year of ICI treatment (20), respectively.

**Table 2:** Characteristics of patients with myocarditis under ICI treatment.

N=116	
Gender	n (%)
Male	77(66.4)
Female	39(33.6)
Primary cancer	n (%)
Lung Cancer	31(26.7)
Melanoma	22(19.0)
Esophageal/gastric cancer	8(6.9)
Thymoma	8(6.9)
Uroepithelial carcinoma	6(5.2)
Kidney Cancer	9(7.8)
Liver Cancer	4(3.4)
Breast Cancer	3(2.6)
Tissue, liposarcoma	3(2.6)
Bone/spinal cord tumor	3(2.6)
Bladder Cancer	2(1.7)
Gallbladder Cancer	3(2.6)
Cervical Cancer	2(1.9)
Mesothelioma	2(1.9)
Colorectal cancer	3(2.6)
Nasopharynx, skin and others	7(6.0)
ICI	n (%)
Pembrolizumab (PD-1)	32(30.2)
Nivolumab (PD-1)	25(23.6)
Nivolumab+Ipilimumab (PD-1+CTLA-4)	21(19.8)
Camrelizumab (PD-1)	6(5.7)
Sindilimab (PD-1)	10(9.4)
Durvalumab (PD-L1)	2(1.9)
Sindilimab+Anlotinib (PD-1+TKI)	1(0.9)
Sindilimab+Lenvatinib (PD-1+ RTK)	1(0.9)
Camrelizumab+Bevacizumab (PD-1+VEGF)	1(0.9)
Cemiplimab (PD-1)	1(0.9)
Durvalumab+Tremelimumab (PD-L1+CTLA-4)	1(0.9)
Toripalimab (PD-1)	1(0.9)
NA	4(3.8)
Median time of onset, day (min–max) [IQR]	22.5(3-275) [16-52]
Best response to ICI	n (%)
CR	6(5.2)
PR	6(5.2)
SD	43(37.1)
PD	40(34.5)
NA	21(18.1)
Grading of Myocarditis	n (%)
G1	7(6.0)
G2	39(33.6)
G3	43(37.1)
G4	27(23.3)
Treatment	n (%)
Glucocorticoids	50(43.1)
Glucocorticoids+Chemical drugs	12(10.3)
Glucocorticoids+ Immunoglobulins	10(8.6)
Glucocorticoids+Life-Support	6(5.2)
Glucocorticoids+Chemical drugs+Immunoglobulins	6(5.2)
Glucocorticoids+Immunoglobulins+Life-Support	4(3.4)
Glucocorticoids+Biologics	3(2.6)
Glucocorticoids+Plasma exchange	6(5.2)
Glucocorticoids+Chemical drugs+Plasma exchange	3(2.6)
Glucocorticoids+Immunoglobulins+Plasma exchange	4(3.4)
Glucocorticoids+Chemical drugs+Life-Support	3(2.6)
Glucocorticoids+Immunoglobulins+Plasma exchange+Life-Support	2(1.7)
Glucocorticoids+Chemical drugs+Biologics+Life-Support	1(0.9)
Glucocorticoids+Immunoglobulins+Biologics	1(0.9)
Glucocorticoids+Biologics+Life-Support	1(0.9)
Glucocorticoids+Plasma exchange+Life-Support	1(0.9)
Immunoglobulins+Plasma exchange	1(0.9)
Glucocorticoids+Chemical drugs+ Biologics	1(0.9)
Glucocorticoids+Biologics+Chemical drugs	1(0.9)
Outcome	n (%)
Recovery	26(22.4)
Improvement	35(30.2)
Death	55(47.4)
Management of ICI	n (%)
Treatment already completed at the onset	12(10.3)
Continued	14(12.1)
Temporarily discontinued, then restarted	11(9.5)
Permanently discontinued	48(41.4)
Not reported	31(26.7)
ICI treatment line	n (%)
First-line	33(28.4)
Second-line	54(46.6)
Third-line	8(6.9)
Fourth-line	1(0.9)
Multi-line	1(0.9)
NA	19(16.4)
Associated irAEs	n (%)
Yes	64(55.2)
No	52(44.8)
Response to myocarditis treatment	n (%)
Improved	50(43.1)
Not improved	40(34.5)
NA	36(31.0)

Of the 116 included patients, the majority were male (66.4%) and received PD-1/PD-L1 inhibitors as monotherapy (75.0%). The three most common primary tumors were lung cancer (26.7%), melanoma (19.0%), and esophageal/gastric cancer (6.9%). In addition, ICIs are usually used as first/second-line (75.0%) treatment. Following ICI treatment, most patients exhibited stable disease (SD, 37.1%) or progressive disease (PD, 34.5%). ICI-associated myocarditis was severe or life-threatening (G3 or G4) in most cases (60.4%), with only a small proportion of patients (6.0%) experiencing grade 1 myocarditis. Given that myocarditis is a fatal adverse event, the mortality rate was 47.4% among 116 cases. Only 26 cases of recovery (22.4%) and 35 cases of improvement (30.2%) related to this condition were reported. We also summarized the main characteristics of 11 Cases of ICI-associated myocarditis in cancer patients treated and rechallenge with ICIs (**Table 3**).

**Table 3:** 11 Cases of ICI-associated myocarditis in cancer patients treated and rechallenge with ICIs.

Author, year	Age, sex	Tumor type	ICI Type	ICI target	myocarditis onset time	myocarditis diagnosis	ICI-related myocarditis Grade	myocarditis symptom	Tumor progression	outcome	Treatment to death time	ICI Discontinuation	Treatment method	myocarditis Outcome
Shindo, 2022 (21)	79, Male	Stomach Cancer	PD-1	Nivolumab	11days	Confirmation of diagnosis (2)	Grade 2	Muscle weakness of lower limbs appeared in 11 days	PD	Death by disease progression	2 months	temporarily discontinued	glucocorticoid	Recovery/NA
Gallegos, 2019 (22)	47, Female	Metastatic melanoma	CTLA-4+PD-1	ipilimumab and nivolumab	120days	Confirmation of diagnosis (2)	Grade 4	Low plasma replacement pressure, ventricular tachycardia, pulmonary edema	PD	Death	7 days	temporarily discontinued	glucocorticoid	Death/7 days
Shen, 2021 (23)	53, Female	Thymoma	PD-1	Pembrolizumab (200 mg)	21 days	Confirmation of diagnosis (2)	Grade 2	Cough, chest tightness, muscle weakness, fatigue	PD	Recovery	NA	temporarily discontinued	glucocorticoid	Recovery/6 months
Kee, 2022 (24)	69, Male	Lung cancer	PD-1	Pembrolizumab	23days	Suspected diagnosis (3)	G3	exertional dyspnea and orthopnea,left eye ptosis	SD	death	163days	temporarily discontinued	Glucocorticoids+Chemical drugs+Life-Support+Immunoglobulins	Death/163days
Zhang, 2022 (25)	68, Female	thymoma	PD-1	Camrelizumab (200 mg, 1/21d)	11days	Suspected diagnosis (3)	G3	dyspnea, fatigue, and poor appetite,palpitation, and poor appetite	SD	death	5days	temporarily discontinued	glucocorticoid+ immunoglobulin+chemical drug	Death/5days
Wintersperger, 2022 (26)	52, Male	Melanoma	PD-L1	PD-L1	21days	Confirmation of diagnosis (2)	Grade 3	Weakness, shortness of breath	SD	Improved	NA	temporarily discontinued	Glucocorticoids + biologics + chemical drugs	Recovery/44days
Lie, 2020 (27)	79,Male	Malignant pleural mesothelioma (MPM)	PD-1	Nivolumab (3 mg/kg)	42days	Confirmation of diagnosis (2)	G3	proximal limb and truncal weakness, dyspnea and generalized fatigue	CR	recovery	NA	temporarily discontinued	Glucocorticoids+Chemical drugs	Recovery/90days
Bawek, 2021 (28)	68, Male	melanoma	PD-1	Nivolumab	21days	Possible diagnosis (3)	G2	shortness of breath, intermittent palpitations, dizziness, and nausea	SD	death	14days	temporarily discontinued	glucocorticoid	Death/14 days
Delombaerde, 2022 (29)	69, Male	Metastatic bile duct cancer	PD-1	nivolumab (3 mg/kg) and ipilimumab (1 mg/kg)	21days	Suspected diagnosis (3)	Grade 2	Episodes of low retrosternal epigastric pain without dyspnea, palpitations, nausea, or stool changes after 1 d	SD	Improved	NA	temporarily discontinued	glucocorticoid	Improved/2 weeks
Zhou, 2022 (30)	67, Male	Squamous cell carcinoma of the lung	PD-L1	Durvalumab	7 days	Possible diagnosis (3)	Grade 3	Fever, breathing difficulties	PD	Improved	7 days	temporarily discontinued	Glucocorticoids+Chemical drugs	Recovery/7 days
Hardy, 2020 (31)	81, Male	RCC metastasis	CTLA-4+PD-1	Iipilimumab and nivolumab	21 days	Probable diagnosis (3)	G4	fatigue, decreased appetite, and weight loss.	NA	Death	2days	temporarily discontinued	Glucocorticoids+ plasma exchange	Death/2 days

Single glucocorticoid agents were administered to 43.1% of the patients, whereas most patients (56.9%) received a combination of glucocorticoids and other therapies. Methylprednisolone was the most frequently administered glucocorticoid, accounting for 43.1% of the cases. The most common methylprednisolone schedule was 1–2 mg/kg/day. Additionally, combination strategies involving glucocorticoids with other therapeutic agents, such as chemical drugs (10.3%), biologics (2.6%), life support (5.2%), immunoglobulins (8.6%), or plasma exchange (5.2%), have also been applied to treat patients with myocarditis of different severities. At the onset of myocarditis, 12 patients (10.3%) had already completed all ICI treatments, and only 14 (12.1%) continued ICIs, whereas 59 (50.9%) discontinued ICIs temporarily (11 patients) or permanently (48 patients). After re-challenge with ICIs, only one of the 11 patients experienced myocarditis recurrence.

Patients with complete response/partial response (CR/PR) usually exhibited a higher improvement rate (83.3%) than patients with SD/PD (55.4%). Of the 116 cases included, myocarditis development was followed by continuation of ICIs in 14 (continued after evaluation) and 11 (temporarily discontinued, then restarted) cases, corresponding to an oncologic efficacy of CR (4), PD (8), PR (2), SD (7), NA (4), and a favorable outcome of 52% (13/25) comparable to the outcome of overall ICI treatment of healing and improvement (52.6%). The occurrence of myocarditis did not affect the efficacy of immunosuppressive therapy. In clinical practice, physicians need to carefully and adequately assess the benefit-risk ratio of patients before initiating ICI therapy and after myocarditis before deciding whether to rechallenge ICI. Patients with myocarditis treated with glucocorticoids had a better improvement rate (62.0%) than those who did not receive hormones. Notably, compared with ≤G3 myocarditis patients, G4 patients have higher improvement rate at 59.3%, although these associations were not statistically significant (**Table 4**).

**Table 4:** Association between characteristics of patients and myocarditis outcome.

Characteristics	Outcome	P
**Grade at the onset**	Improvement rate, % (n/N)	
G4(N=27)	59.3(16/27)	0.2637
≤G3(N=89)	50.6(45/89)	
**Best response to ICI**	Improvement rate, % (n/N)	
CR/PR(N=12)	83.3(10/12)	0.1140
SD/PD(N=83)	55.4(46/83)	
**Glucocorticoids**	Improvement rate, % (n/N)	
Yes (N=50)	62.0(31/50)	0.1348
No(N=66)	45.5(30/66)	
**Glucocorticoids+Chemical drugs**	Improvement rate, % (n/N)	
Yes (N=12)	50.0(6/12)	>0.9999
No(N=104)	52.9(55/104)	
**Glucocorticoids+ Immunoglobulins**	Improvement rate, % (n/N)	
Yes(N=10)	70(7/10)	0.3287
No(N=106)	50.9(54/106)	
**Discontinuation of ICI**	Improvement rate, % (n/N)	
YES(N=59)	61.0(36/59)	0.0575
No(N=26)	34.6(9/26)	
**Rechallenge of ICI**	Recurrence rate, % (n/N)	
YES(N=11)	22.2(2/9)	0.0226
No(N=48)	0(0/48)	

## Discussion

The largest number of published case reports on myocarditis in patients with cancer treated with ICIs were included and analyzed in this article. We presented the main characteristics of the 106 patients and found associations between some patient characteristics and myocarditis outcomes.

Based on our results, male sex, lung cancer, melanoma, and treatment with anti-PD-1, anti-PD-L1, or anti-PD-1 in combination with CTLA-4 may increase the risk of ICI-associated myocarditis. Previous studies have suggested that the combination of anti-CTLA-4 and anti-PD-1 is one of the strongest risk factors for ICI-associated myocarditis. The pharmacovigilance data indicated that a 4.74-fold higher risk of myocarditis than nivolumab alone (32). Our research showed that patients receiving anti-CTLA-4 and anti-PD-1 antibodies may exhibit a higher grade of myocarditis, with 46% incidence of grade 4 myocarditis. Another large retrospective pharmacovigilance study revealed that patients with myocarditis are more often male (66%), having melanoma (40.7%) or lung cancer (32%), and are treated with anti-PD-1/PD-L1 as a single agent (69%) (12). Consistent with these data, patients included in our article were mostly male patients receiving anti-PD-1/PD-L1 antibodies for melanoma or lung cancer. Genetic variations, including somatic or germline tumors, may also contribute (33). Furthermore, clinical trials involving a large number of patients are required to identify predisposing factors for myocarditis and other ICI-associated cardiovascular toxicities.

The exact incidence of myocarditis in patients with cancer treated with ICIs remains unknown. ICI-based cancer trials in the early time did not prospectively screen for myocarditis (34). Current investigations have reported that the incidence rates range from 0.1% to 1.14% across different series (13, 32). This broad range may be attributed to heterogeneity, such as the different grades of severity of the cases and the diverse distribution of potential risk factors for ICI-associated myocarditis (35). In addition, because of the difficulty of myocarditis diagnosis cases in these trials might have been missed. Overall, the true incidence of ICI-associated myocarditis may be higher, and further prospective trials should focus on this issue.

ICI-associated myocarditis represents a clinically unmet problem because it may be fatal. The mortality rates range from approximately 35.8%, as reported in our analysis, to >50%, as reported in a previous study (13). To date, no international consensus has been reached covering ICI-associated myocarditis screening, surveillance, prevention, and treatment. The diagnosis of myocarditis can be challenging in clinical settings, particularly in patients receiving ICIs. In current clinical practice, ICI-associated myocarditis is often a multipronged diagnosis of exclusion, ruling out other causes of symptomatology (for example, cancer progression and acute coronary syndrome), and includes a comprehensive analysis of cardiac imaging, biomarker tests, and endomyocardial biopsy (36). Based on a multicenter study from American College of Cardiology (13), Mahmood et al. (13) proposed that the traditional diagnostic pathway of is the observation of new-onset cardiovascular symptoms in patients receiving ICI therapy, further laboratory and imaging tests, and medical consultations, ultimately leading to a diagnosis of ICI-associated myocarditis. Another expert guideline from European Society of Cardiology (14) indicated that the initial diagnosis of ICI-associated myocarditis relies on the identification of aberrant cardiovascular symptoms, a recent elevation in troponin levels, the presence of new electrocardiogram (ECG) abnormalities, and urgent cardiovascular imaging to other causes of myocardial injury, such as acute coronary syndrome. In fact, most patients exhibit clinical symptoms suggestive of ICI-associated myocarditis, elevated troponin levels, and/or an abnormal baseline ECG (37). However, increased serum troponin concentrations are difficult to interpret in asymptomatic patients, which highlighted improved predictive biomarkers are needed. Cardiac magnetic resonance imaging (MRI) can be used for further diagnosis (38). In clinical practice, an endomyocardial biopsy has traditionally been regarded as the gold standard for myocarditis diagnosis (39). The histopathological characteristics of ICI-associated myocarditis involve infiltration of T lymphocytes (both CD4+ and CD8+), macrophages, and myocyte death, whereas B lymphocytes are not observed (32). However, as an endomyocardial biopsy is an invasive examination, it poses a psychological burden on patients. In the future, prospective multi-institutional studies are needed to explore effective non-invasive examinations, such as predictive biomarkers and medical imaging, for the screening and surveillance of patients.

The clinical implications of ICI-associated myocarditis vary among studies. Patients with fulminant myocarditis exhibit early symptoms after ICI treatment, including arrhythmias/conduction disturbances, dyspnea, concomitant skeletal myositis, and myasthenia gravis (12). This was consistent with our results which showed that dyspnea was found in 31% of patients. Another study highlighted that the concomitant presence of skeletal myositis and myasthenia gravis following after ICI treatment should increase awareness of myocarditis (40). Our research indicated that 20% of patients with myocarditis also exhibited skeletal myositis or myasthenia gravis. In contrast to fulminant cases, “smoldering” cases of ICI-associated myocarditis have also been documented (35, 41). However, no studies have revealed the long-term consequences of ICI-associated myocarditis. Therefore, given the growing number of cancer survivors receiving ICIs, understanding the long-term cardiovascular effects of ICIs is a future challenge for oncologists and cardiologists.

Treatments for ICI-associated myocarditis have been largely extrapolated from amount of irAEs therapies, including cessation of ICIs, glucocorticoids, chemical drugs, and supportive management (42). For myocarditis, higher initial steroid doses (e.g., intravenous methylprednisolone, 1 g/day) have been suggested (43). In the present review, almost all patients (98.2%) received glucocorticoids and achieved a 15.1% recovery rate and a 35.8% improvement rate, suggesting that glucocorticoids are the cornerstone of ICI-associated myocarditis treatment. Nonetheless, the findings of our analysis revealed that the mortality was substantial (50.8%). In addition to glucocorticoids, various case reports have demonstrated the efficacy of other medications such as tacrolimus (44), mycophenolate mofetil (45), abatacept (46), and alemtuzumab (47). Although these treatments are classified as immunosuppressive modalities, their specific mechanisms of action differ (48). For example, abatacept is a soluble protein composed of the CTLA-4 extracellular domain fused to the Fc region of IgG, which limits the costimulatory signals of T cells (49). Wei et al. explored whether abatacept could ameliorate the disease progression of ICI-associated myocarditis in a mouse model and mitigate its fulminant course in patients (50). Further it is necessary of prospective clinical trials to compare single or combination efficacy with that of other therapies.

Considering steroids as the main treatment for immune myocarditis, we also summarized new immune checkpoint inhibition into the biologic agent category in **Table 2**, including six case-use reports of infliximab, one case of anti-thymocyte globulin (ATG), and one case of abatacept. Two studies reported nonsignificant improvement in symptoms related to myocarditis with infliximab (51, 52), while three cases reported a worsening manifestation of symptoms related to myocarditis with infliximab (53–55), and another study reported the use of infliximab but not describing the results (26). One study found the use of ATG was suspended due to poor patient status (56), and another study reported the myocarditis symptoms were improved with the use of Abatacept (57). Although new immune checkpoint inhibitors have been recommended as second-line therapy for immune myocarditis after steroid resistance, and although this study summarized case reports on immune myocarditis in the last five years, there is uncertainty about the efficacy of biologics such as tumor necrosis factor-α antagonists, ATG, and abatacept in actual case reports due to the lack of prospective studies, and this may be related to our limitations of the collected cases.

The guidelines of the American Society of Clinical Oncology for the management of irAEs in patients treated with ICIs (42) recommend the early use of high doses of glucocorticoids (e.g., methylprednisolone 1 g/day) and a combination of mycophenolate, antithymocyte globulin, or infliximab for the treatment of refractory and recurrent myocarditis. Conversely, although some experts have advocated TNF-α antagonists (such as infliximab) for ICI-associated myocarditis, concerns have been raised regarding their application in patients with heart failure (58).

Furthermore, we also compared our results with current known cohorts of ICI-associated myocarditis (11–13, 59) (**Table 5**). Our results were almost consistent with other four cohorts. Based on the known cohorts, the incidence rate of ICI-associated myocarditis is ranged from 0.39%-1.14%, representing a small entity of ICI-associated adverse events. However, the fatal rate is as high as 39.7%-50%, and our results indicate the fatal rate is 47.4%, indicating the unmet clinical need of ICI-associated myocarditis. Besides, the combination of anti-PD-1/PD-L1 and anti-CTLA-4 seems to cause higher fatal rate compared with single use of ICIs. Therefore, in clinical practice, physicians need to carefully and adequately assess the benefit-risk ratio of patients before initiating ICI therapy and after myocarditis before deciding whether to rechallenge ICI.

**Table 5:** The comparison of this study with current known cohort.

	Wang’s study (59)	Moslehi’s study (11)	Mahmood’s study (13)	Salem’s study (12)	Our study
Incidence rate	131(0.42%) of 31,059 cases	NA	11(1.14%) of 964 cases	122(0.39%) of 31,321 cases	NA
Timing (median, range)	32 days (3-355)	27 days (5-155)	34 days (21-75)	30 days (1-240)	22.5 days (3-275)
Fatality rate	52 (39.7%) of 131 cases	46 (46%) of 101 cases	NA	61 (50%) of 122 cases	55 (47.4%) of 116 cases
Anti–PD-1/PD-L1 deaths	27 (8%) of 333 cases	22 (36%) of 61 cases	NA	40 (44.4%) of 90 cases	42 (52.5%) of 80 cases
Anti–CTLA-4 deaths	3 (2%) of 193 cases	3 (60%) of 5 cases	NA	NA	NA
Combination PD-1/CTLA-4 deaths	22 [25%] of 87 cases	18(67%) of 27 cases	NA	21 (65.6%) of 32 cases	12 (54.5%) of 22 cases

**Figure 2:**
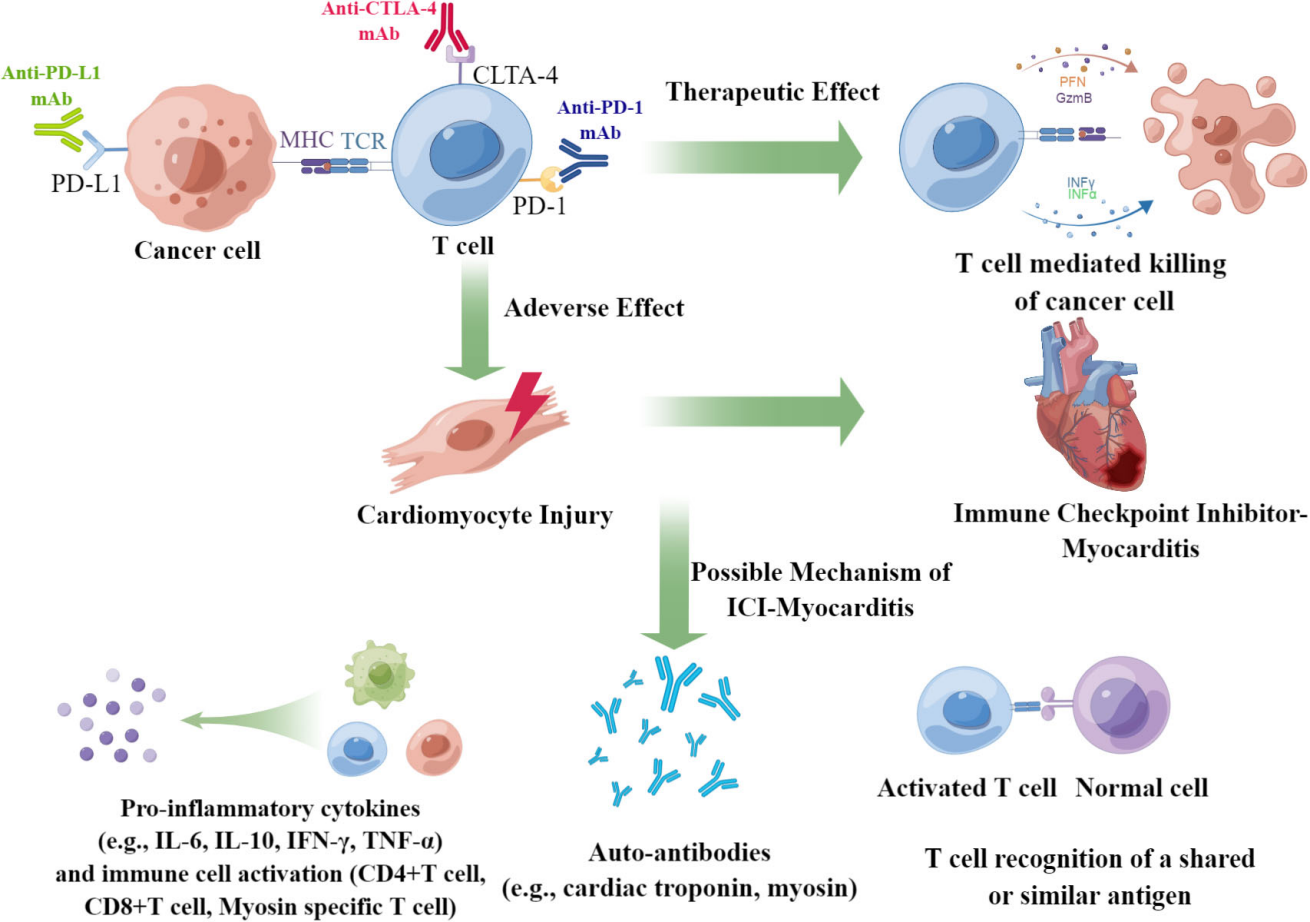
Summary of the underlying mechanisms of ICI-associated myocarditis. ICI-associated myocarditis is a serious adverse events of patients of cancers received ICI treatment. The possible mechanism of ICI-associated myocarditis may be due to the elevation of pro-inflammatory cytokines, emergence of auto-antibody, and the T cell recognition of a shared or similar antigen.

The exact mechanism of the pathogenesis of ICI-associated myocarditis remains unclear, and some concerns should be addressed in future studies (60) (**Figure 2**). First, we precisely determined the incidence of ICI-associated myocarditis. The potential lethality of cardiotoxicity limits the clinical application of ICIs. Given the apparent low frequency (<1%) of ICI-associated myocarditis, one would not anticipate this possibility, if not for the high death rate (35.8%, as reported in our analysis) associated with this adverse event. The inconsistent morbidity and mortality rates of ICI-associated myocarditis reflect an unmet clinical need; therefore, prospective studies should be performed to address this issue. Second, studies are needed to identify predictive markers and medical imaging technologies for patients with high-risk ICI-associated myocarditis, and an endomyocardial biopsy is always required for the final diagnosis. Third, more multicenter clinical trials necessary for formulating and standardizing diagnostic and therapeutic schemes. Further studies should focus on the relative balance between potentially disturbing the cancer treatment and alleviating cardiotoxicities. The most important issue is understanding the pathogenesis of ICI-associated myocarditis at the molecular and cellular levels. Some questions should be addressed: How do ICIs affect immune-cardiac interrelationships? What cardiac antigens are inciting? Why do self-antigens elicit harmful immune responses? Is cell death a critical process in pathogenesis, which cell death patterns are involved if it was true? Does the predominance of arrhythmias primarily reflect disturbances in the conduction system of the heart, or is it generalized by systematic inflammation? Taking into account the greater complexity of human, studies involving the blood and tissues obtained from patients are critical for understanding these mechanisms.

Study limitations. The current investigation also had several limitations, primarily attributable to the retrospective nature of case reports: (1) case reports are inherently subjective, which provide a non-random sample, and often do not allow for causal inferences; (2) although multiple databases were search, publication bias was not entirely ruled out, and mild cases could have been under-reported; particularly, only a few G1 myocarditis cases have been reported; (3) some detailed information on risk factors, diagnostics or management of myocarditis could be missing; (4) patients selected for re-challenge of ICIs were probably those in better clinical condition, and in clinical practice, the decision of re-challenge should be considered carefully on a case-by-case basis; (5) the sample size included in this article was limited and relied on the literature of a small collection of case reports which was not allowed for a more comprehensive quantitative analysis. Furthermore, the associations observed between the patient characteristics and outcomes were not statistically significant, rendering our findings speculative.

## Conclusions

ICI-associated myocarditis is an emerging clinical concern that has attracted the attention of cardiologists and oncologists. To integrate information on ICI-associated myocarditis, we recovered and analyzed the largest number of published case reports in our work. A reasonable workflow to manage ICI-associated myocarditis was proposed based on this article as follows: for severe cases (G3 or G4), discontinuation of ICIs, administration of high-dose glucocorticoids (methylprednisolone 1 g/day) and other drugs, plasma exchange, and life support measures; for moderate cases (G1 or G2), discontinuation of ICIs and administration of regular-dose glucocorticoids (methylprednisolone 1-2 mg/kg/day). Once full recovery or improvement is achieved, steroids must be adjusted to low doses (prednisone <10 mg/day) or discontinued. Moreover, re-challenge with ICIs appears feasible in selected patients based on the decisions made by the cardiovascular physician, oncologist, and patient.

## Data availability statement

The original contributions presented in the study are included in the article/**Supplementary Material**. Further inquiries can be directed to the corresponding authors.

## Ethics statement

This study did not require ethical approval.

## Author contributions

CW: Data curation, Investigation, Methodology, Writing – original draft. GZ: Data curation, Formal analysis, Investigation, Writing – original draft. ZZ: Data curation, Investigation, Writing – original draft. LY: Data curation, Writing – original draft. SL: Data curation, Writing – original draft. GL: Data curation, Writing – original draft. HW: Data curation, Writing – original draft. JH: writing – original draft. SW: Supervision, Writing – review & editing. NL: Conceptualization, Funding acquisition, Supervision, Writing – review & editing.
